# Rapid Sanger Sequencing of the 16S rRNA Gene for Identification of Some Common Pathogens

**DOI:** 10.1371/journal.pone.0088886

**Published:** 2014-02-14

**Authors:** Linxiang Chen, Ying Cai, Guangbiao Zhou, Xiaojun Shi, Jianhui Su, Guanwu Chen, Kun Lin

**Affiliations:** 1 Department of Preventive Medicine, Shantou University Medical College, Shantou, Guangdong, China; 2 Laboratory of Molecular Biology, Shantou Entry-Exit Inspection and Quarantine Bureau, Shantou, Guangdong, China; Kliniken der Stadt Köln gGmbH, Germany

## Abstract

Conventional Sanger sequencing remains time-consuming and laborious. In this study, we developed a rapid improved sequencing protocol of 16S rRNA for pathogens identification by using a new combination of SYBR Green I real-time PCR and Sanger sequencing with FTA® cards. To compare the sequencing quality of this method with conventional Sanger sequencing, 12 strains, including three kinds of strains (1 reference strain and 3 clinical strains, which were previously identified by biochemical tests), which have 4 *Pseudomonas aeruginosa*, 4 *Staphyloccocus aureus* and 4 *Escherichia coli,* were targeted. Additionally, to validate the sequencing results and bacteria identification, expanded specimens with 90 clinical strains, also comprised of the three kinds of strains which included 30 samples respectively, were performed as just described. The results showed that although statistical differences (P<0.05) were found in sequencing quality between the two methods, their identification results were all correct and consistent. The workload, the time consumption and the cost per batch were respectively light versus heavy, 8 h versus 11 h and $420 versus $400. In the 90 clinical strains, all of the *Pseudomonas aeruginosa* and *Staphyloccocus aureus* strains were correctly identified, but only 26.7% of the *Escherichia coli* strains were recognized as *Escherichia coli*, while 33.3% as *Shigella sonnei* and 40% as *Shigella dysenteriae*. The protocol described here is a rapid, reliable, stable and convenient method for 16S rRNA sequencing, and can be used for *Pseudomonas aeruginosa* and *Staphyloccocus aureus* identification, yet it is not completely suitable for discriminating *Escherichia coli* and *Shigella* strains.

## Introduction

To date, conventional Sanger sequencing technology is sometimes used in a few diagnostic laboratories, however, it remains time-consuming and laborious. In this article, we have improved the conventional Sanger sequencing and validated it for detecting and genotyping the most common pathogens, including *Pseudomonas aeruginosa*, *Staphyloccocus aureus* and *Escherichia coli*. We presented this protocol and it described a new combination of SYBR Green I real-time polymerase chain reaction (PCR) and Sanger sequencing of DNA collected and extracted through *Whatman* FTA® cards. The bacterial 16S ribosomal RNA gene was used for PCR amplification and subsequent sequencing. Sample collection and DNA preparation for PCR in this assay involve directly use of FTA® cards instead of commercial kits, boiling, phenol-chloroform extraction and ethanol precipitation, or also using FTA® cards but should be prior cleaned with purification reagent or sterile water in previous studies [1]–[4]. *Whatman* FTA® paper is a commercial product that provides a remarkably easy way to collect, preserve and purify genomic DNA from bacteria, consisting of filter paper impregnated with a proprietary mix of chemicals that serve to lyse cells, prevent the growth of bacteria, protect the DNA in the sample, and can be stored at room temperature for even as long as 50 years [5]. Though it has been widely used for PCR, few researches reported its utility of pathogens sequencing typing, we would give a confirmation here. The common 16S rRNA sequencing technique in diagnostic laboratories is still currently based on the conventional Sanger sequencing method, called “first generation sequencing”, involving PCR amplification, product qualitative detection and separation by gel electrophoresis, purification of the amplicon through ethanol precipitation, sequencing by an amplification reaction and final capillary electrophoresis. Due to time-consuming, laborious, high operation skills requirement and potential hazard of *ethidium bromide* in agarose gel electrophoresis, the first generation sequencing technique has not been commonly used in most diagnostic laboratories [6]. To save time and reduce workload, we make improvement and propose a new combined protocol involving direct sequencing of the product generated by diagnostic SYBR Green?real-time PCR. The PCR product is diagnosed via the amplifying curve, and specificity of the product is determined by analysis of the melting curve, avoiding the step of agarose gel electrophoresis. In addition, we optimized all hands-on instrument steps by using modern reagents, by means of sequencing 16S rRNA gene of reference and clinical pathogenic strains, we validated the applicability and also found the shortcomings of 16S rRNA gene sequencing method for identification.

## Materials and Methods

### Ethics Statement

The study protocol was approved by the Human Ethical Committee of Shantou University Medical College and Shantou Central Hospital, China. The patient records/information was anonymized and de-identified prior to analysis.

### 1 The Evaluation of Improved Sanger Sequencing and Compared to Conventional Sanger Sequencing with Small Samples

#### 1.1 Tested strains

To save time and cost for comparing these two methods, we only target 12 pathogenic strains, including 3 reference strains, ATCC.27853 *Pseudomonas aeruginosa* (American Type Culture Collection), AS.26003 *Staphylococcus aureus* and AS.44113 *Escherichia coli* (China General Microbiological Culture Collection Center), and 9 clinical isolates (had been identified by using conventional biochemical tests by microbiologists), each of 3 *Pseudomonas aeruginosa*, 3 *Staphylococcus aureu* and 3 *Escherichia coli*.

#### 1.2 Preparation of bacterial suspension and DNA processed in each method

The clinical bacterial strains were isolated and the reference strains were rejuvenated. Both of them used conventional cultural methods, then the suspensions of pathogen strains were made at proper concentrations. DNA prepared for improved method was performed referred to Menassa et al. [7]. In brief, after vortexing thoroughly, 50 microliters of suspension were dropped onto a FTA® card and were allowed to permeate evenly through the paper. All cards were then allowed to air-dry at room temperature so as to inactivate pathogens by the reagents within the cards. For conventional method, DNA was processed as Corless et al. [8] described but needs some modification, briefly, pipetting all the bacterial suspensions each of 100 µl to 900 µl sterile distilled water, centrifugation at 12,000×g for 3 min prior to remove the 900 µl supernatant, repeating this step one more time and the residual 100 µl mixture which contains bacteria were boiled at 100°C for 10 min to release DNA, after slightly centrifugation, the supernatant can be stored at 4°C and prepared for PCR using.

#### 1.3 SYBR Green ? Real-time 16S rDNA PCR by improved method

Punch one disk with appropriate diameter from the sample spot on the FTA® card and place the disk into a real-time PCR reaction tube for direct SYBR Green?PCR, which contained 10 µl of SYBR Green?PCR Master Mix reagent (Takara), 1 µl each of 2 µM stocks of universal bacteria 16S rRNA gene forward and reverse primers (forward: 5′-TGGA GAGTTTGATCCTGGCTCAG-3′; reverse: 5′-TACCGCGGCTGCTGGCAC-3′) [9], and 9 µl of water. PCR was performed in an Roche LightCycler 480 system thermocycler (Roche) with an initial step of 2 min at 95°C, followed by 35 cycles of 10 s at 95°C, 20 s at 60°C, and 40 s at 72°C. Fluorescent signal intensities were recorded during the end of the elongation phase in each cycle. To interpret the data, the Cp (cross point) values for each sample and negative control were calculated by using analysis mode of “Abs Quant/2^nd^ Derivative Max”. Besides, we regarded the results as potentially positive (amplified products existed) if the Cp value cycle of amplifying curves was <30, while the melting curve of the amplicon presented a single melting peak. If there were two or more melting peaks in a melting curve, the products would be considered impure and unreliable. Specially, to seek for the optimum size of FTA® card disk for PCR in this assay, we used three sizes, which were 0.5-mm, 1.2-mm and 2.0-mm of FTA® of AS.26003 *Staphylococcus aureus* strains for direct PCR. Furthermore, to quest the best bacteria concentration dropping onto the FTA® card, a standard curve, including a linear range of known quantification from 6×10^4^ to 6×10^9^ CFU ml^−1^ of AS.26003 *Staphylococcus aureus* strains, was constructed. Thereafter formal experiments started when the above-mentioned optimal condition had been affirmed.

#### 1.4 Traditional PCR and products qualitative detection through agarose gel electrophoresis by conventional method

The processed DNA extraction was placed in PCR tubes, with the following components added and the final volume adjusted to 20 µL with sterile double distilled water: 100 nM each primer, 800 µM dNTPs, 1.5 mM MgCl_2_&2.5 U Taq polymerase. Using Roche LightCycler 480 the specimens were heated to 96°C for 10 min followed by 35 cycles of 96°C for 10 s, 59°C for 20 s, 72°C for 30 s, with a final extension step of 72°C for 10 min. The reaction products were held at 4°C until use within 24 h. The PCR products were visualised using a 1.5% agarose gel with ethidium bromide staining. A DNA marker of known DNA fragment sizes (2000 bp ladder) was run along side the specimens to aid in identification of the products. Electrophoresis in Tris-borate-EDTA buffer was performed at 100 V for 40 minutes, and photographed under UV light illumination (UNIVERSAL HOOD.365 nm), when visual band were observed at about 500 bp fragment, PCR succeeded.

#### 1.5 Processing of PCR products and subsequent sequencing PCR by two methods

In order to eliminate potentially adverse effects of PCR reagents on subsequent Sanger sequencing, we diluted the PCR reaction both of two methods 5-fold by adding 2 µl PCR product to 8 µl water. Then sequencing PCR reaction was performed in a 20 µl final volume containing 4 µl of BigDye Terminator v3.1 Sequencing Buffer (5×) (Applied Biosystems), 2 µl of 1 µM amplify PCR backward primer, 4 µl of BigDye Mix, 9 µl water and 1 µl of diluted PCR product,and run in a Veriti 96-Well Fast Thermal Cycler (Applied Biosystems) using the following parameters: denaturation at 98°C for 2 min, followed by 25 cycles of 96°C for 10 s, annealing at 50°C for 5 s and extension at 60°C for 4 min.

#### 1.6 Purification of sequencing PCR products and capillary electrophoresis by two methods

In the improved method, Sanger sequencing products was purified using the BigDye XTerminator purification kit (Applied Biosystems). SAM solution (45 µl) and BigDye XTerminator solution (10 µl) were respectively added and premixed in each 0.2 ml tube. Then 5 µl Sanger sequencing product was added in each tube, vortexed for 5 min, centrifuged at 2000×g for 2 min. Then 10 µl supernatant in each tube was transferred into a plate and covered with septa. After a pulse spin, the plate was mounted in a 3130 Genetic Analyzer (Applied Biosystems) using default module BDx_StdSeq50_POP7_1 optimized to a 3 s injection. Then the sequences were automatically compiled using Sequencing Analysis 5.3.1 software (Applied Biosystems). While in conventional method, Sanger sequencing products were purified by using traditional ethanol precipitations referring to Peattie [10]. Briefly, added 35 µl 100% ethanol solution and 2 µl to each 0.2 ml tube which contains 5 µl products, centrifuged at 12000×g for 20 min then carefully discarded all the supernatants. Added 50 µl 75% ethanol solution to each tube to further eliminate impurities, discarded all the supernatants after 2 min. Air-dried products in the tubes for 20 minutes. Added 10 µl Hi-Di™ Formamide to each tube and vortex as needed, then centrifuged at 2000×g for 1 min and following a final step of denaturation at 96°C for 3 min. All the 10 µl mix in each tube was transferred into the plate and processed as improved method described.

#### 1.7 Nucleotide blast analysis in the Genbank database for species or genus identification in two methods

Sequences obtained were blasted with the GenBank database (http://www.ncbi.nlm.nih.gov/. 16S Ribosomal RNA Sequences (Bacteria and Archaea)) for species or genus assignment. The highest identity was selected as the identified species or genus.

### 2 Improved Sequencing Protocol for Practical Application with Clinical Samples

90 pathogen strains, comprised of 30 samples each of *Pseudomonas aeruginosa*, *Staphyloccocus aureus* and *Escherichia coli*, were isolated from in-patients admitted to the Shantou Central Hospital (Shantou, China) between May 2012 and July 2012, and identified at the species level also using conventional culture and phenotypic methods by microbiologists before PCR and sequencing. And all the following procedures were performed as the section of improved method in 2.1.2?2.1.7 described.

## Results

### Optimized Tests of Improved Sanger Sequencing Protocol

**Figure 1:**
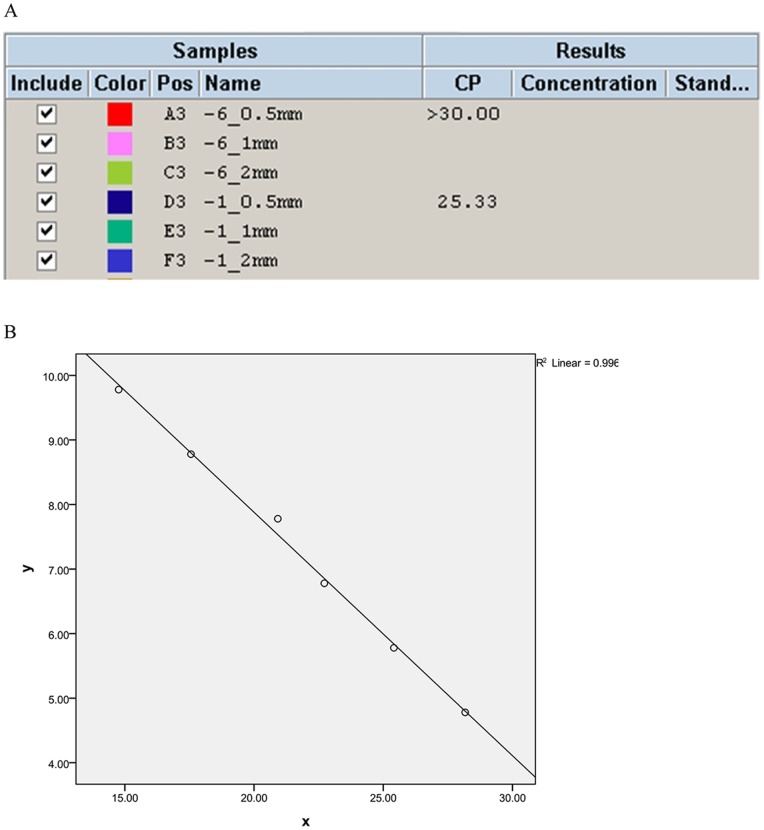
A: Three sizes of diameter punch (0.5-mm, 1-mm, 2-mm) involving higher and lower bacteria concentrations for PCR. −6 stands for 60 CFU ml^−1^, −1 stands for 6×10^6^ CFU ml^−1^; B: Standard curve of real-time quantitative PCR of 6 decimal dilutions of representative Staphylococcus aureus ranging from 6×10^4^ to 6×10^9^ CFU ml^−1^.

In our optimized text, we found that no matter whether 1.2-mm and 2.0-mm disks were dropped with either a higher concentration or a lower concentration of suspension, neither of them could produce an interpretable Cp value in amplification curves, it suggesting 0.5-mm was the most suitable option (Figure 1A). When a series of known quantification from 6×10^4^ to 6×10^9^ CFU ml^−1^ in 0.5-mm card of AS.26003 *Staphylococcus aureus* strains were built to SYBR Green I PCR, a linear relationship between the Cp and the logarithm of concentration was observed (Figure 1B). The amplification efficiency calculated from these data was 1.98, very close to the theoretical maximal yield 2. The slope of the standard curve is −0.37, and the correlation coefficient is 0.97, generating a regression equation Y(Log concentration) = -0.37X(Cp) +15.442. According to the Cp values and regression equation, we suggest that the best concentration on 0.5-mm FTA® disk should range from 6×10^4^ to 6×10^7 ^CFU ml^−1^, for the corresponding Cp values were from 20.92 to 28.17. However, either higher or lower concentrations are not recommended, since higher concentration is difficult to prepare and easy to generate cross-contamination, while lower concentration is not sufficient to be amplified.

### Comparison Results from 12 Specimens by Using the Two Methods

**Figure 2:**
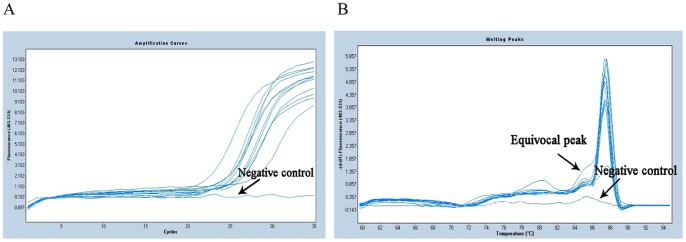
Amplification curves (A) and melting curves (B) of 12 specimens and negative control from improved method.

**Figure 3:**
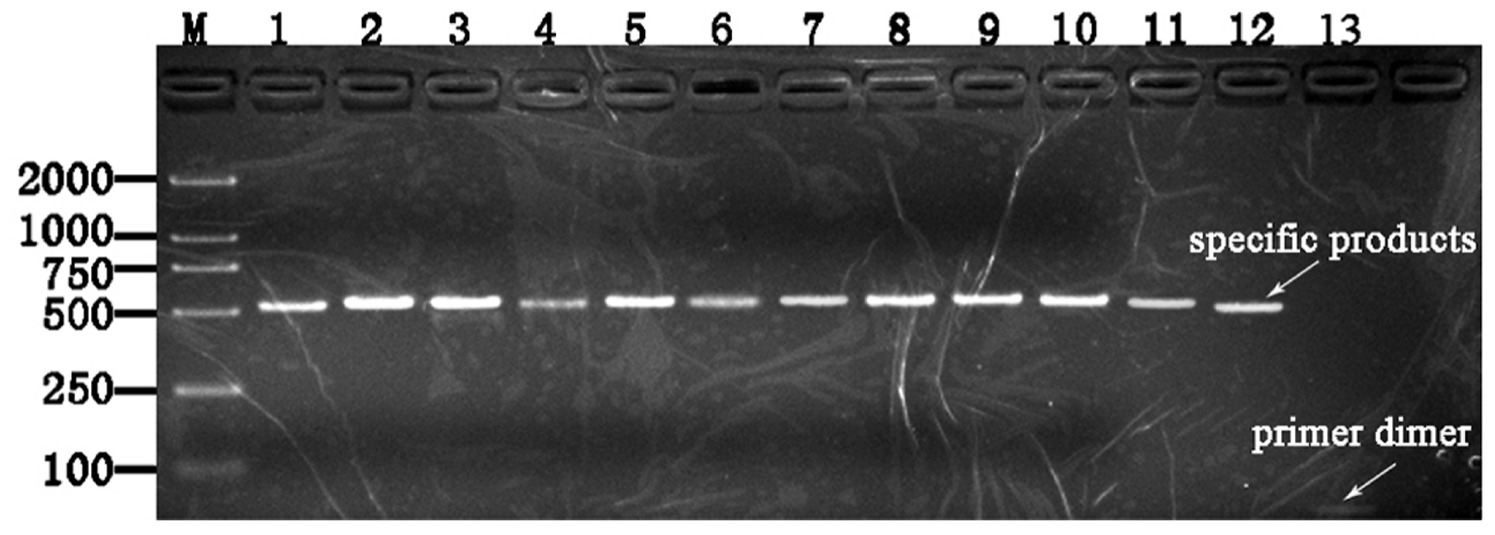
Ethidium-bromide–stained agarose gel of PCR reaction products from 12specimens through conventional method. Lane M, 100-base-pair (bp) DNA ladder; lane 1, reference strain of *Escherichia coli*; lane2, reference strain of *Pseudomonas aeruginosa*; lane 3, reference strain of *Staphylococcus aureus*; lane 4 to lane 12, representative clinical strain of *Escherichia coli 1-3*, *Staphylococcus aureus 1-3* and *Pseudomonas aeruginosa 1-3*; lane 13, negative control without DNA template.

**Figure 4:**
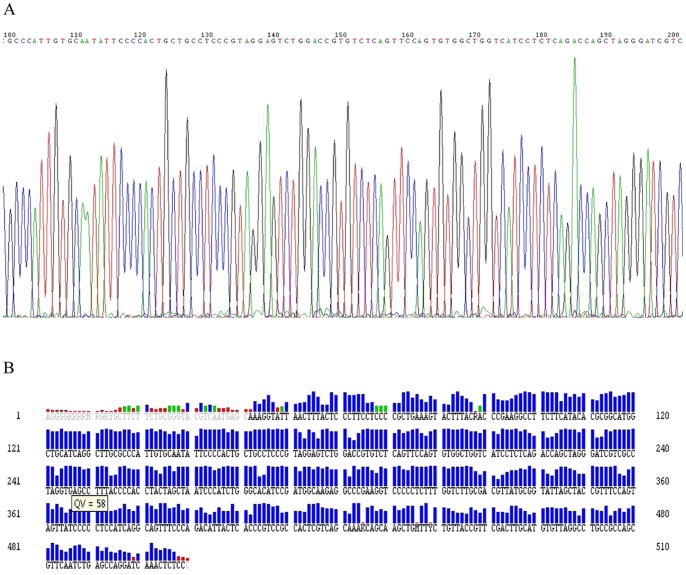
Sequence chromatogram (A) and sequence quality evaluation (B) from clinical Staphylococcus aureus strain 1. Red stands for low quality, green for medium quality, blue for high quality.

In this improved method, after the first PCR step, the amplification curves and melting curves of all 12 strains were showed in Figure 2. The curves showed that all strains were performed perfectly, as amplification curves presented Cp value <30, and the melting curves presented a single sharp peak. Instead, the negative control (PCR mix excluding DNA) appeared as a line in which no Cp value was observed. Sometimes, equivocal peaks were observed in curves, but non adverse impact would affect the identified results. In conventional method, PCR products formation was visually confirmed through agarose gel electrophoresis (Figure 3). From lane 1 to lane 12, the specific about 500-bp products were detected and presented an unambiguous visible band in each lane as we expected. In lane 13, it was noteworthy that a faint band was observed and located at about 20 bp fragment, and we confirmed that this minute quantity of amplicon was probably universal primers dimmer, however, it could not be sequenced by itself, and also did not interfere with the16S rRNA fragment and subsequent sequencing. Finally, all the 12 specimens in two methods were successfully sequenced and presented explicit chromatograms for unambiguously distinguishing (the representative diagram of sequence chromatogram and quality referred to Figure 4). In order to evaluate the sequencing quality, we analysed their detailed qulity parameters through DNA Sequencing Analysis software 5.1 (Applied Biosystems). According to the manufacturer’s instructions, KB basecaller of sequence generates QV from 1 to 99, with typical high quality bases scoring from 20 and above, typical medium quality bases ranging from 15 to 19, while low quality base scoring less than 15. The LOR (length of read), which were defined as the usable range of high-quality or high-accuracy base sequence, determined by quality values, were 477.4 and 477.8. The average base numbers with low QV were respectively 66.9 and 46.3, high QV were 414 and 444.3. Herein we defined two derived parameters: PLQ (percentage of low QV bases) as percentage of base numbers with low QV to LOR, and PHQ (percentage of high QV bases) as percentage of base numbers with high QV to LOR. The average PLQs of the two methods were 14.0% and 9.69%, average PHQs were 86.7% and 93.0%, sample score (the average quality value of the bases in the clear range sequence for that sample) were 34.1 and 38.1. Statistical texts (using Wilcoxon Matched-Pairs Signed-Ranks Test or Matched-Pairs t-text) showed that all the differences had statistical significance (P<0.05) in PLQ, PHQ and sample score, and we considered that the sequences quality from conventional method was superior to the improved method (Table 1). However, even so, they had no impact on identification results when submitted to Genbank for blasting, in other word, although statistical significance was found in comparison of sequences quality, the blasting results from two methods were still correct and consistent, which respectively 99% or 100% matched the three kinds of strains recorded as NR_026078.1/, NR_037007.1/and NR_074891.1/from NCBI. Notably, the 6^th^ sample *Escherichia coli* had another more similar matching item NR_074894.1 (Table 2), and we would give explanations below.

**Table 1:** Sequence quality from the 12 samples in two methods.

Samples description	Improved Sanger sequencing/Conventional Sanger sequencing					
	LOR	Low QV (base numbers)	PLQ (percentage of low QVs.%)	High QV (base numbers)	PHQ (percentage of high QVs.%)	Sample score
1. AS.44113 *Escherichia coli*	463/492	84/37	18.1/7.5	360/454	77.8/92.3	27/37
2. ATCC.27853 *Pseudomonas aeruginosa*	466/466	78/39	16.9/8.4	401/436	86.1/93.6	34/42
3. AS.26003 *Staphylococcus aureus*	501/469	51/39	10.2/8.3	433/437	86.4/93.2	38/34
4. *Escherichia coli 1*	490/479	56/47	11.4/9.8	432/450	88.2/93.9	35/37
5. *Escherichia coli 2*	469/491	85/53	18.1/10.8	401/449	85.5/91.4	36/38
6. *Escherichia coli 3*	476/476	96/36	20.2/7.56	394/460	82.8/96.6	36/42
7. *Staphylococcus aureus 1*	487/478	56/75	11.5/15.7	427/401	87.8/83.9	31/30
8. *Staphylococcus aureus 2*	488/486	68/47	13.9/9.67	418/441	85.7/90.7	29/32
9. *Staphylococcus aureus 3*	500/464	37/57	7.4/12.3	470/437	94.0/94.2	37/34
10. *Pseudomonas aeruginosa 1*	482/469	60/48	12.5/10.2	432/449	89.6/95.7	37/43
11. *Pseudomonas aeruginosa 2*	485/478	51/44	10.5/9.2	434/456	89.5/95.4	34/43
12. *Pseudomonas aeruginosa 3*	422/485	81/33	19.2/6.8	366/462	86.3/95.3	35/45
Population mean	477.4/477.8	66.9/46.3	14.0/9.7	414/444.3	86.7/93.0	34.1/38.1
Statistical text. P value	–	–	<0.05*	–	<0.05*	<0.05#

^*^using Wilcoxon Matched-Pairs Signed-Ranks Test.

# using Matched-Pairs t-text.

**Table 2:** Sequencing results of the 12 specimens separately performed by two methods.

Reference strains				Sequence with highest blastn scores	
Description (cases)	Source	(average) Valid Sequence Length (after manually called) improved method/conventional method	%identity improved method/conventi-onal method		Accessions/Description
Reference strains					
ATCC.27853 *Pseudomonas aeruginosa*	American Type Culture Collection	411/422	99%/99%		NR_026078.1/*Pseudomonas aeruginosa*
AS.26003 *Staphylococcus aureus*	China General Microbiological Culture Collection Center	408/420	100%/100%		NR_037007.1/*Staphylococcus aureus*
AS.44113 *Escherichia coli*		394/410	99%/99%		NR_074891.1/*Escherichia coli*
Clinical strains					
*Pseudomonas aeruginosa* (3)	urine, pus, sputum or faeces from in-patient	400/412	99%/99%		NR_026078.1/*Pseudomonas aeruginosa*
*Staphylococcus aureus* (3)		412/422	100%/100%		NR_037007.1/*Staphylococcus aureus*
*Escherichia coli* (2)		395/405	99%/99%		NR_074891.1/*Escherichia coli*
*Escherichia coli* (1)		401/414	99%/99%		NR_074894.1 *Shigella sonnei*

### Other Miscellaneous Comparison of Two Sequencing Protocols

For the 12 specimens (Table 3), the successful rate for my first experiments was 100%, of course, it should be caution enough for operator performing, especially in conventional method. The workload, time consumption, and the cost per batch with 12 samples were respectively light versus heavy, 8 h versus 11 h and $420 versus $400. Obviously, it was more labor-saving and time-saving if using improved Sanger sequencing, while an advantage in conventional Sanger sequencing was that it cost less. However, we would rather recommend the former method than the latter, which was an inconvenient job indeed.

**Table 3:** Comparison of several items between two methods with 12 specimens.

	Comparison items			
Methods	successful rate (once experiment)	workload	Time consumption per batch	Cost per batch
Improved Sanger sequencing	100%	Light	8 h	$420
Conventional Sanger sequencing	100%	Heavy	11 h	$400

### Results of 90 Clinical Isolates by Using the Improved Sequencing Protocol

**Figure 5:**
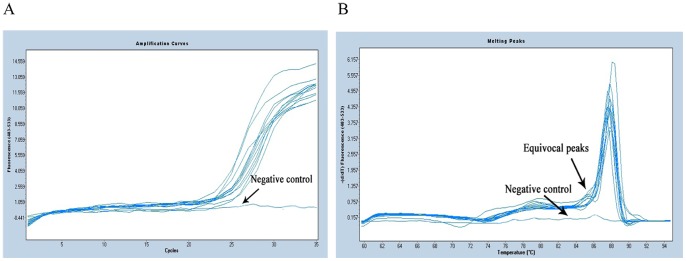
Amplification curves (A) and melting curves (B) of partial experimental strains.

Among the 90 real-time PCR amplifications performed on the experimental isolates, all amplification curves were considered as positive with Cp values ranged from 20.15 to 29.55 (mean: 25.12). From the 90 melting curves, 70 showed a single peak with a Tm value of 88°C as reference strains’, so the corresponding products were regarded as the purest products and were the most suitable for subsequent sequencing. The other 20 showed dual peaks including a high main peak with a Tm of 88°C, and a minor equivocal peak with Tm of approximate 85°C (Figure 5). Since the equivocal peaks were much smaller than the main peaks, we considered that the amount of “unknown products” was far less than the 16S rRNA fragment products, and inferred they would not interfere with the following sequencing process [11]. Furthermore, after agarose gel electrophoresis, the signals produced by “unknown products” were relatively weak or were only seen in the absence of DNA as input.

After screening based on their amplification curves, all of the clinical samples entered into a rapid and simplified sequencing pipeline. 90 PCR products were ultimately sequenced and gave valid read length between a minimum of 367 nucleotides and a maximum of 481 nucleotides, with a mean ± standard deviation length of 404.5±20.5 bases, and some ambiguous bases that could be manually called. Besides, the sequence quality value showed that average base numbers with low QV were 68.3, high QV were 420, LOR were 475.6, PLQ were 14.4%, PHQ were 88.4% and sample score was 35.2≥20, ≤1% probability of a miscalled base. According to Tewari D et al. [12], isolates were categorized into either a species with ≥99% match, a genus with ≥95% match, or a higher taxon with <95% having criteria described previously. Therefore, in 90 experimental samples, identification of *Pseudomonas aeruginosa* and *Staphylococcus aureus* to the species level of the best match in the Genbank database corresponded to 100% of the organisms identified by conventional microbiological methods. But from 30 *Escherichia coli* samples, the best matching strains with significant alignment, respectively were 8 *Escherichia coli* (26.7%), 10 *Shigella sonnei* (33.3%) and 12 *Shigella dysenteriae* (40%) (Table 4).

**Table 4:** Identification results of clinical pathogen strains.

Species identified by conventional techniques (strain numbers)	Valid Sequence Length (after manually called)	Sequence with highest blastn scores	
		%identity	Accessions/Description
*Pseudomonas aeruginosa* (30)	383–412	99%–100%	NR_074828.1 *Pseudomonas aeruginosa*
*Staphylococcus aureus* (30)	386–481	99%–100%	NR_037007.1 *Staphylococcus aureus*
*Escherichia coli* (8)	396–415	99%	NR_074891.1 *Escherichia coli*
*Escherichia coli* (10)	367–412	99%	NR_074894.1 *Shigella sonnei*
*Escherichia coli* (12)	371–477	99%	NR_074892.1 *Shigella dysenteriae*

**Figure 6:**
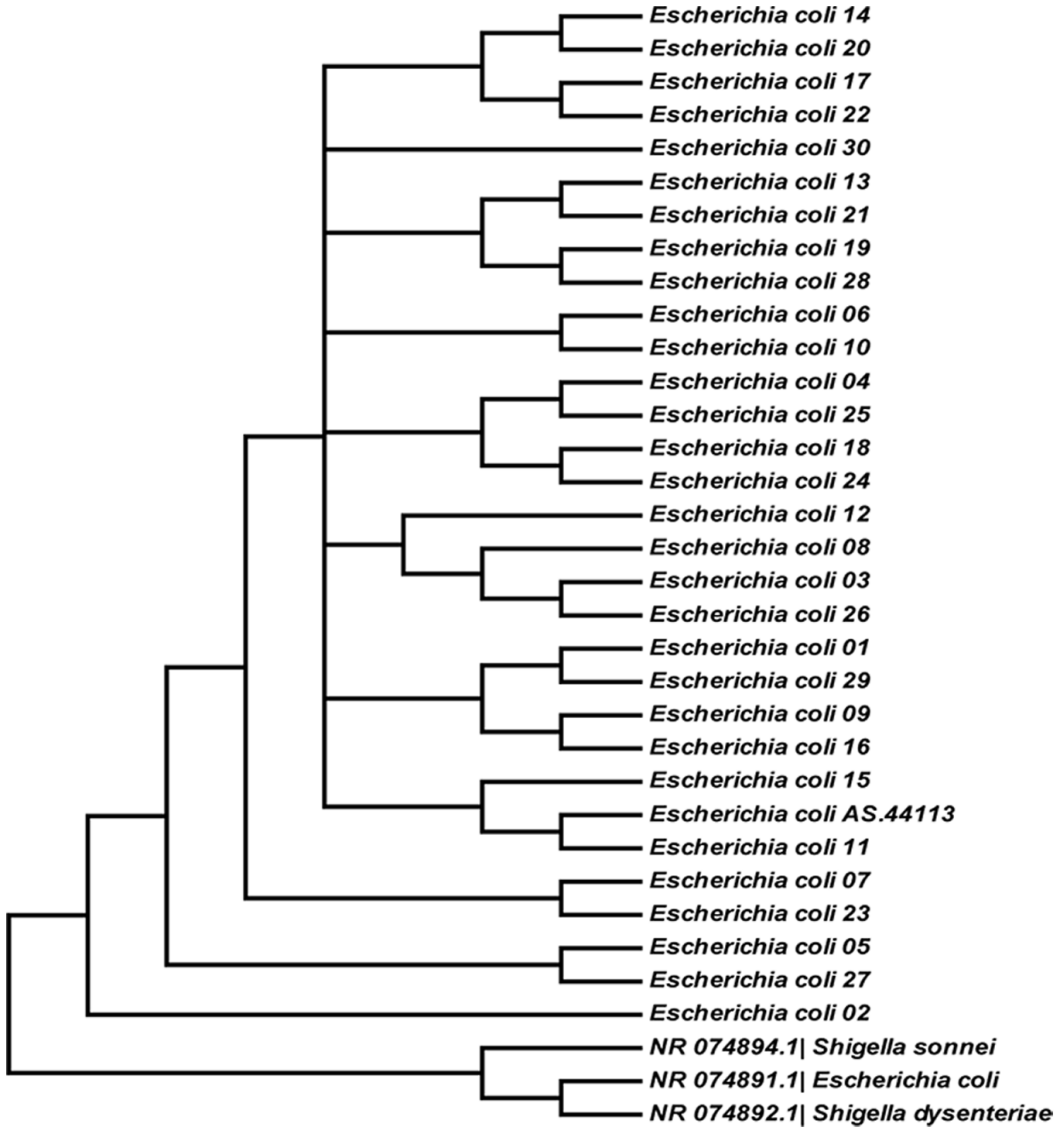
Dendrogram showing the phylogenetic relationships among *Escherichia coli* sequences based on 16S rDNA partial genes. The tree was constructed by using the neighbor-joining method with Mega 5.02.

Specially, for the 30 specimens of *Escherichia coli* strains with discordant blasting, sequence similarity would be assessed further by constructing a relatedness diagram (phylogenetic tree) using a minimum of 1,000 bootstrap trees. 31 *Escherichia coli* sequences (30 clinical specimens and 1 reference specimens) and 3 corresponding best matching sequences (*Escherichia coli*, NR_074891.1; *Shigella sonnei*, NR_074894.1; *Shigella dysenteriae*, NR_074892.1) from Genbank would join the construction (Figure 6). It is noticeable that in this phylogenetic tree, the 3 sequences from Genbank were too similar to be separated, and were unable to get close to those specimens that were respectively matched, and compared to the previous study using 16S rRNA gene sequence to construct Phylogenetic tree between *Shigella* and *Escherichia coli* [13], the giving results verified the results of our experiment.

## Discussion

In recent years, instead of traditional biochemical methods, Sanger sequencing is becoming increasingly popular [14], however, the traditional processes are still relatively troublesome, but these drawbacks will be overcame after our improvement. In the comparative test, when evaluating the sequences quality of both methods, with parameters of PLQ, PHQ and sample score, statistical differences were found, we submitted all the sequences to NCBI for blasting, however, the highest blastn scores in two methods were equal and the identification results were both correct and consistent. These convincing data have illustrated the utility of the improved Sanger sequencing we proposed. In addition, the turnaround time required for real-time PCR, Sanger sequencing, capillary electrophoresis reaction and data analysis plus labor consumption needed to complete 500 bp sequencing in improved method which was less than 8 h per batch, including 12 specimens, while additional 3 h per batch should be provided in the latter method. The cost per batch was $36 for DNA captured by FTA® card, and $384 for amplification reagents, sequencing, purification of products and capillary electrophoresis (excluding instrument and labor costs), while it would cost less in the latter method due to avoiding some reagent. However, much more time and labor should be needed during the procedure of DNA extraction, agarose gel electrophoresis, and products purification. Even worse, in the processing of agarose gel electrophoresis, we were unable to prevent the toxicity of ethidium bromide which is a kind of strong carcinogen. While SYBR Green I added in PCR reaction was able to check the effectiveness of PCR safely, save time and reduce workload as well. The DNA templates for PCR, FTA® card with bacterial suspension was directly PCR amplified in improved method, while DNA extracting from bacterial suspension in the latter method, no doubt, the former choice was a more convenient method, and it also reduced the risks of microbial contamination. In the step of products purification, improved method just needed reagent for simple mix and slight centrifugation, while the extraordinary laborious operation, such as oft-repeated high speed centrifugation and oft-repeated removing supernatant carefully, were essential in the conventional method.

Additionally, we had applied expanded specimens to assess the utility of our new improved method. SYBR Green ? releases intense fluorescence only when combined with double-stranded DNA, but does not emit detectable fluorescence, which were detected upon DNA denaturation, it is a non-specific indicator dye [15]. For this reason, the combination of primers and SYBR Green ?leads to some equivocal melting curves, but their Cp values still remain at an acceptable level, and agarose gel electrophoresis results of the corresponding products also emerged as a well-defined pattern of bands, so we still have sequenced them. Though, compared to others, the final 20 chromatograms appeared to be devoid of some additional discernible bases, with a QV larger than 20, high-quality sequences were still acquired, and matches were still obtained when submitted to the Genbank blast system, supporting the report that some interference within products was not completely eliminated or impacted by primer formation [16].

From the identification results of pathogenic strains, we learn that partial 16S rRNA gene sequencing is a suitable tool for *Staphylococcus aureus* and *Pseudomonas aeruginosa* identification, which have produced consistent results with conventional culture methods as others have done [17], [18]. However, 30 *Escherichia coli* specimens generated 3 blast results of *Shigella sonnei*, *Shigella dysenteriae* and *Escherichia coli*, and the 16S rDNA-based phylogenetic tree suggested that it was difficult to distinguish each of them. It has been demonstrated by other researchers that there are many similarities in many respects between some *Shigella* (e.g. *Shigella dysenteriae*) and *Escherichia coli* (e.g. Enteroinvasive *Escherichia coli*), such as clinical symptoms, biochemical characteristics and antigens [19]. In fact, previous study showed that a few *Escherichia coli* have been assigned to a different genus (e.g., *Shigella flexneri*), based primarily on their distinct clinical presentation and their importance as human pathogens [20]. A research by Pupo et al. [21], analyzing sequence variation in housekeeping genes, also showed that most *Shigella* serotypes fall into three clusters within *Escherichia coli*, proving that, it is indeed difficult to distinguish *Shigella* from *Escherichia coli*. So the false identification results in some *Escherichia coli* of our specimens may attribute to the false classification of *Escherichia coli* sequences, which were virtually *Shigella* sequences submitted to GenBank by other researchers.

Compared with conventional Sanger sequencing, our improved protocol has emerged as a faster and more convenient method to identify those common bacteria. However, it also should be applied cautiously. Firstly, although sequencing is particularly helpful in situations where organisms are difficult to characterize by using conventional culture methods, but 1 to 14% of the isolates remain unidentified after testing [22]. Secondly, the variable regions, as a foundation for discriminating bacteria, only distributing V1–V3 in the first 500 bp area, is one third of full-length of 16S gene (V1–V10) [23]. This system uses universal primers to amplify and sequence a 500 bp fragment from the 5′-terminus of the 16S rRNA gene [24], but only a mean of 404 bp is read, because the first approximately 100 bp had to be manually discarded owing to residual SYBR Green?left over from PCR products, and was difficult to be removed by purification kit. Consequently the V1, distributed in the first 104 bp, have to be discarded and hence slightly impaired the discrimination ability of the sequencing chromatogram. Lastly, though SYBR Green?does not require specific probes to be developed, as is the case for some other detection chemistries. However, the detection specificity of SYBR green I assays depends entirely on the PCR primers [25], suggesting that it is important to ensure the high specificity of primers, so negative control in PCR reaction should be needed.

In the future, 16S rRNA gene sequencing will continue to be the gold standard for identification of most bacteria [26], and better automation of such an improved technology may put it into routine use in large microbiology laboratories. The assay described here is a suitable tool for sequencing identification of *Pseudomonas aeruginosa* and *Staphyloccocus aureus* faster and more conveniently, but it is not completely accurate to discriminate *Escherichia coli* and *Shigella* strains. Under optimal conditions, the protocol can be applied for any PCR and sequence-based analysis after proper modification. The time-consumption and the cost remain acceptable for most laboratories, and will become further reduced as the technology becomes more widely adopted and refined. However, despite the fact that it is difficult to accurately assign some particular isolates to a specific species, assigning to a certain genus can successfully assist the further research [27].

## References

[1] Gustavsson I, Lindell M, Wilander E, Strand A, Gyllensten U (2009) Use of FTA card for dry collection, transportation and storage of cervical cell specimen to detect high-risk HPV. Journal of Clinical Virology 46: 112–116. 19628427 10.1016/j.jcv.2009.06.021

[2] Mullen M, Howard D, Powell R, Hanrahan J (2009) A note on the use of FTA™ technology for storage of blood samples for DNA analysis and removal of PCR inhibitors. Irish Journal of Agricultural and Food Research: 109–113.

[3] Schuurman T, De Boer RF, Kooistra-Smid A, van Zwet AA (2004) Prospective study of use of PCR amplification and sequencing of 16S ribosomal DNA from cerebrospinal fluid for diagnosis of bacterial meningitis in a clinical setting. J Clin Microbiol 42: 734–740. 14766845 10.1128/JCM.42.2.734-740.2004 PMC344470

[4] Smith L, Burgoyne LA (2004) Collecting, archiving and processing DNA from wildlife samples using FTA® databasing paper. BMC ecology 4: 4. 15072582 10.1186/1472-6785-4-4 PMC406513

[5] Aye KS, Matsuoka M, Kai M, Kyaw K, Win AA, et al (2011) FTA card utility for PCR detection of Mycobacterium leprae. Jpn J Infect Dis 64: 246–248. 21617312

[6] Fukushima H, Tsunomori Y, Seki R (2003) Duplex real-time SYBR green PCR assays for detection of 17 species of food- or waterborne pathogens in stools. J Clin Microbiol 41: 5134–5146. 14605150 10.1128/JCM.41.11.5134-5146.2003 PMC262470

[7] Menassa N, Bosshard PP, Kaufmann C, Grimm C, Auffarth GU, et al (2010) Rapid detection of fungal keratitis with DNA-stabilizing FTA filter paper. Invest Ophthalmol Vis Sci 51: 1905–1910. 19850832 10.1167/iovs.09-3737

[8] Corless CE, Guiver M, Borrow R, Edwards-Jones V, Kaczmarski EB, et al (2000) Contamination and sensitivity issues with a real-time universal 16S rRNA PCR. J Clin Microbiol 38: 1747–1752. 10790092 10.1128/jcm.38.5.1747-1752.2000 PMC86577

[9] Hall L, Doerr KA, Wohlfiel SL, Roberts GD (2003) Evaluation of the MicroSeq system for identification of mycobacteria by 16S ribosomal DNA sequencing and its integration into a routine clinical mycobacteriology laboratory. J Clin Microbiol 41: 1447–1453. 12682128 10.1128/JCM.41.4.1447-1453.2003 PMC153882

[10] Peattie DA (1979) Direct chemical method for sequencing RNA. Proc Natl Acad Sci U S A 76: 1760–1764. 377283 10.1073/pnas.76.4.1760 PMC383470

[11] Han XY, Pham AS, Tarrand JJ, Sood PK, Luthra R (2002) Rapid and accurate identification of mycobacteria by sequencing hypervariable regions of the 16S ribosomal RNA gene. Am J Clin Pathol 118: 796–801. 12428802 10.1309/HN44-XQYM-JMAQ-2EDL

[12] Tewari D, Cieply S, Livengood J (2011) Identification of bacteria recovered from animals using the 16S ribosomal RNA gene with pyrosequencing and Sanger sequencing. Journal of Veterinary Diagnostic Investigation 23: 1104–1108. 22362789 10.1177/1040638711425583

[13] Fukushima M, Kakinuma K, Kawaguchi R (2002) Phylogenetic analysis of Salmonella, Shigella, and Escherichia coli strains on the basis of the gyrB gene sequence. J Clin Microbiol 40: 2779–2785. 12149329 10.1128/JCM.40.8.2779-2785.2002 PMC120687

[14] Killgore G, Thompson A, Johnson S, Brazier J, Kuijper E, et al (2008) Comparison of seven techniques for typing international epidemic strains of Clostridium difficile: restriction endonuclease analysis, pulsed-field gel electrophoresis, PCR-ribotyping, multilocus sequence typing, multilocus variable-number tandem-repeat analysis, amplified fragment length polymorphism, and surface layer protein A gene sequence typing. J Clin Microbiol 46: 431–437. 18039796 10.1128/JCM.01484-07 PMC2238077

[15] Willi B, Meli ML, Luthy R, Honegger H, Wengi N, et al (2009) Development and application of a universal Hemoplasma screening assay based on the SYBR green PCR principle. J Clin Microbiol 47: 4049–4054. 19828748 10.1128/JCM.01478-09 PMC2786680

[16] Vliegen I, Jacobs JA, Beuken E, Bruggeman CA, Vink C (2006) Rapid identification of bacteria by real-time amplification and sequencing of the 16S rRNA gene. J Microbiol Methods 66: 156–164. 16371239 10.1016/j.mimet.2005.11.005

[17] Becker K, Harmsen D, Mellmann A, Meier C, Schumann P, et al (2004) Development and evaluation of a quality-controlled ribosomal sequence database for 16S ribosomal DNA-based identification of Staphylococcus species. J Clin Microbiol 42: 4988–4995. 15528685 10.1128/JCM.42.11.4988-4995.2004 PMC525259

[18] Bhattacharya D, Sarma PM, Krishnan S, Mishra S, Lal B (2003) Evaluation of genetic diversity among Pseudomonas citronellolis strains isolated from oily sludge-contaminated sites. Appl Environ Microbiol 69: 1435–1441. 12620826 10.1128/AEM.69.3.1435-1441.2003 PMC150093

[19] Li Y, Liu D, Cao B, Han W, Liu Y, et al (2006) Development of a serotype-specific DNA microarray for identification of some Shigella and pathogenic Escherichia coli strains. J Clin Microbiol 44: 4376–4383. 17021058 10.1128/JCM.01389-06 PMC1698391

[20] Lan R, Reeves PR (2001) When does a clone deserve a name? A perspective on bacterial species based on population genetics. Trends Microbiol 9: 419–424. 11553453 10.1016/s0966-842x(01)02133-3

[21] Pupo GM, Lan R, Reeves PR (2000) Multiple independent origins of Shigella clones of Escherichia coli and convergent evolution of many of their characteristics. Proceedings of the National Academy of Sciences 97: 10567–10572. 10.1073/pnas.180094797 PMC27065 10954745

[22] Janda JM, Abbott SL (2007) 16S rRNA gene sequencing for bacterial identification in the diagnostic laboratory: pluses, perils, and pitfalls. J Clin Microbiol 45: 2761–2764. 17626177 10.1128/JCM.01228-07 PMC2045242

[23] Petti CA (2007) Detection and identification of microorganisms by gene amplification and sequencing. Clin Infect Dis 44: 1108–1114. 17366460 10.1086/512818

[24] Cloud JL, Conville PS, Croft A, Harmsen D, Witebsky FG, et al (2004) Evaluation of partial 16S ribosomal DNA sequencing for identification of Nocardia species by using the MicroSeq 500 system with an expanded database. J Clin Microbiol 42: 578–584. 14766819 10.1128/JCM.42.2.578-584.2004 PMC344514

[25] Monnet C, Correia K, Sarthou AS, Irlinger F (2006) Quantitative detection of Corynebacterium casei in cheese by real-time PCR. Appl Environ Microbiol 72: 6972–6979. 16950905 10.1128/AEM.01303-06 PMC1636138

[26] Woo PCY, Ng KHL, Lau SKP, Yip K, Fung AMY, et al (2003) Usefulness of the MicroSeq 500 16S ribosomal DNA-based bacterial identification system for identification of clinically significant bacterial isolates with ambiguous biochemical profiles. J Clin Microbiol 41: 1996–2001. 12734240 10.1128/JCM.41.5.1996-2001.2003 PMC154750

[27] Woo PCY, Cheung EYL, Leung K, Yuen K (2001) Identification by 16S ribosomal RNA gene sequencing of an Enterobacteriaceae species with ambiguous biochemical profile from a renal transplant recipient. Diagn Microbiol Infect Dis 39: 85–93. 11248520 10.1016/s0732-8893(01)00206-1

